# Digital twin-assisted edge-local query resolution for privacy–utility trade-off in location-based services

**DOI:** 10.1371/journal.pone.0360633

**Published:** 2026-10-15

**Authors:** Mohammed Hasan A., Nemi Chandra R.

**Affiliations:** 1 Department of Computer Science, Central University of South Bihar, Gaya, Bihar, India; 2 Department of Computer Science, University of Lahej, Sabir, Lahej, Yemen; The Pennsylvania State University, UNITED STATES OF AMERICA

## Abstract

Location-Based Services (LBS) generate large volumes of sensitive, location-dependent queries that must be processed under strict and often conflicting constraints of privacy and utility. Existing differential privacy (DP) mechanisms face a fundamental limitation: strong guarantees inject excessive noise and degrade utility, while utility-oriented methods under-protect users. We propose DT-ELR (Digital Twin-assisted Edge-Local Resolution), a framework that jointly optimises disclosure and retrieval utility through two tightly integrated algorithms and an on-device digital twin. Algorithm 1 (SensitivityClassifier) calibrates a monotone geo-indistinguishability budget from a data-derived sensitivity score, validated against a semantic venue-category labelling (AUC 0.81). The digital twin predicts from on-device signals alone whether releasing a query would improve its answer (AUC 0.90); self-sufficient queries are resolved against an on-device venue cache with no release and no budget spent. Algorithm 2 (ClosedFormAllocator) then distributes the remaining budget across released queries in closed form, matching the exact dynamic-programming optimum to within 1%. Experiments on three public check-in corpora — Foursquare-NYC, Gowalla and Brightkite — using an end-to-end nearest-venue retrieval task and three implemented adversaries, show that DT-ELR reduces optimal-inference localization success on sensitive queries from 61.3% to 11.4% on Foursquare-NYC at a matched mean budget, while raising end-to-end retrieval precision from 0.244 under full release to 0.559. Twin-guided release routing outperforms a tuned distance heuristic at every matched release rate, approaching an oracle ceiling of 0.565, and keeps 73% of sensitive queries entirely on the device. Component analysis confirms that each mechanism contributes independently, with edge-local resolution dominant, and the sensitivity model transfers to Gowalla and Brightkite without refitting. Against three recent location-perturbation mechanisms at a matched budget, DT-ELR attains a dominant privacy–utility frontier on all three corpora: higher utility at equal disclosure, and lower disclosure at equal utility.

## 1. Introduction

The rapid proliferation of smartphones and ubiquitous wireless connectivity has transformed Location-Based Services (LBS) into indispensable platforms for real-time location-based services, social query propagation, and peer-assisted content retrieval [1,2]. Unlike conventional online social networks, LBS operate under severe resource constraints — limited battery capacity, intermittent connectivity, and heterogeneous node mobility — while simultaneously handling queries that are inherently privacy-sensitive because they encode user location, social trajectory, and behavioural patterns [3,4]. The challenge of designing a query mechanism that satisfies both strong formal privacy guarantees and high retrieval utility in this constrained environment represents one of the most pressing open problems in mobile computing research [5,6].

Differential Privacy (DP) has emerged as the de facto mathematical framework for quantifying and bounding information leakage in query-answering systems [7,8]. Under ε-DP, the privacy budget ε controls the sensitivity of query outputs to the inclusion or exclusion of any individual record: smaller ε enforces stronger privacy through larger injected noise, which in turn degrades query utility and raises mean squared error [9]. In LBS query mechanisms, this trade-off is particularly acute because queries are geospatially correlated across users — enabling inference attacks that exploit spatial clustering to de-anonymize trajectories even when individual responses are noisily perturbed [10,11]. Furthermore, the energy cost of noise computation and query routing adds a third dimension to this optimization that is routinely overlooked by privacy-first approaches [12].

A second, less-studied dimension of the LBS query problem is where a query is resolved: given a query with known sensitivity, it can be answered locally from an on-device venue cache (no coordinate released, strong privacy) or released to a service (some disclosure, potentially a better answer). This decision depends on whether releasing the query would actually improve the answer returned, together with the sensitivity classification of the query itself [13,14]. No existing work jointly optimizes these two decisions — noise allocation and release routing — in a single, sensitivity-aware framework.

We evaluate DT-ELR against three recent location-privacy mechanisms—PTPPM [15], PAnDA [16], and LR-Geo [17]—together with the planar Laplace geo-indistinguishability reference [18], all assessed through the identical retrieval-and-attack pipeline at a matched mean budget on three public corpora.

The main contributions of this work are:

1. We introduce DT-ELR, a novel digital-twin-assisted framework for edge-local query resolution that places a digital twin predictor at the center of edge-local query resolution, coupling it with differential privacy (DP) budget management for LBS query processing. Against a uniform-budget release at the same mean budget, the framework lowers optimal-inference localization success on sensitive queries from 61.3% to 11.4%.
2. We design a content-aware sensitivity classifier (Algorithm 1) whose score is derived from four re-identification risk proxies and validated against an independent venue-category labelling, reaching an AUC of 0.81. The score drives a monotone geo-indistinguishability calibration in which the released budget is non-increasing in sensitivity by construction.
3. We introduce edge-local query resolution guided by a digital twin. The twin predicts from on-device signals whether a release would improve the answer, and queries it judges self-sufficient are answered from an on-device venue cache at zero budget and zero disclosure. A closed-form allocator (Algorithm 2) distributes the remaining budget across released queries, matching the exact dynamic-programming optimum to within 1%.
4. We evaluate the framework end-to-end on a real nearest-venue retrieval task rather than on proxy scores, against three implemented adversaries: optimal-inference localization, trace re-identification, and mutual-information leakage. The evaluation reports utility and disclosure separately for sensitive and non-sensitive queries, and includes component-contribution and parameter sensitivity analyses.

The remainder of this paper is organized as follows. Section 2 reviews related work on privacy-preserving query mechanisms in Location-Based Services. Section 3 presents the proposed DT-ELR framework, including the system architecture and the formal specification of Algorithm 1 (*DLP-SensitivityClassifier*) and Algorithm 2 (*ClosedFormAllocator*). Section 4 describes the experimental setup, including datasets, evaluation metrics, and implementation details. Section 5 presents and analyzes the experimental results, including comparative, ablation, and convergence studies. Finally, Section 6 concludes the paper and outlines future research directions.

## 2. Related work

Privacy preservation in location-based services (LBS) has attracted significant research attention. Existing approaches span query protection, trajectory privacy, secure computation, and system-level optimization. However, most solutions address isolated challenges, lacking a unified and adaptive framework.

### 2.1. Query privacy and differential privacy mechanisms

Liu et al. [19] introduced a cache-based privacy protection scheme for continuous location queries, where previously queried results are reused to reduce direct exposure to the LBS server, thereby improving efficiency and minimizing redundant interactions. However, despite reducing query frequency, this approach remains vulnerable to inference attacks arising from observable caching patterns. Building on the broader landscape of such techniques, Rasheed et al. [20] provided a comprehensive survey of location-query privacy mechanisms, including k-anonymity, dummy-based methods, and differential privacy, highlighting the fundamental challenge of balancing privacy strength with service utility. To address privacy at a deeper level, Yuan et al. [21] proposed a semantic correlation-based trajectory protection model using differential privacy, where noise is injected according to semantic relationships between locations, enhancing privacy for mobility patterns but at the cost of noticeable utility degradation. In a further step toward mitigating leakage, Li et al. [22] developed a hybrid framework that combines differential privacy with query control to quantify and limit information exposure in range queries. While this approach improves privacy management, it relies on predefined privacy parameters, making it less effective in dynamically adapting to varying query sensitivity and real-time system conditions.

A parallel line of recent work refines the location-perturbation mechanism itself. Cao et al. [15] protect personalised trajectories under differential privacy while accounting for temporal correlation between successive locations, selecting a perturbed point among nearby candidates with probability decaying in distance. Yu et al. [16] address the cost of solving metric-DP optimisation at scale, approximating the release distribution over a reduced anchor set under a probabilistic metric-DP formulation. Qiu et al. [17] confine the release distribution to a locally relevant candidate set, cutting the optimisation domain while preserving the privacy–utility objective. These mechanisms improve how a single coordinate is perturbed, but each still releases every query to the service; none couples the perturbation with a decision about whether a query needs to be released at all. This work adopts all three as release-mechanism baselines and compares them at a matched budget in Section 5.7.

*Limitation Summary: These works rely on static or uniform privacy mechanisms and lack dynamic sensitivity-aware adaptation.*

*Our Advancement: A query sensitivity-aware model that dynamically adjusts privacy mechanisms based on context.*

### 2.2. Cryptographic and secure query processing

Wu et al. [23] proposed a privacy-preserving bilateral matching scheme for mobile social networks, enabling secure multi-receiver matching with revocation capabilities to enhance access control. While the scheme provides strong security guarantees, it incurs significant computational and communication overhead, limiting its practicality in large-scale or real-time environments. Extending the use of cryptographic protection to query processing, Zheng et al. [24] introduced SecSkyline, which supports skyline query execution over encrypted cloud databases, ensuring data confidentiality during processing. However, the reliance on complex cryptographic operations affects scalability and increases response latency. Similarly, Tong et al. [25] developed VPSL, a verifiable privacy-preserving search framework for cloud-assisted IoT systems that guarantees both correctness and privacy of query results through verification mechanisms. Despite these advantages, the additional verification procedures introduce notable latency and computational costs.

*Limitation Summary: Strong security guarantees come at the cost of high computational complexity and lack of real-time adaptability.*

*Our Advancement: A lightweight, adaptive optimization-based framework that balances privacy and efficiency without heavy cryptographic overhead.*

### 2.3. Federated learning and distributed privacy

Zheng et al. [26] presented a comprehensive review of privacy-preserving federated learning in power systems, highlighting the benefits of secure aggregation and decentralized computation. However, they also identified key challenges, including communication overhead and system heterogeneity, which affect scalability and efficiency. To address some of these limitations, Wang et al. [27] introduced a communication-efficient and personalized federated learning model that improves scalability and adapts to user-specific patterns while maintaining privacy guarantees. Despite these advancements, such approaches primarily focus on model training and do not make per-query release decisions or calibrate disclosure to the sensitivity of an individual query.

*Limitation Summary: These approaches focus on model training privacy, not real-time query optimization or sensitivity-aware decisions.*

*Our Advancement: Integration of real-time decision-making using digital twins, enabling adaptive query processing.*

### 2.4. Graph Neural Networks for privacy-aware systems

Gao et al. [28] presented a comprehensive survey of graph neural network (GNN)-based recommender systems, demonstrating their effectiveness in modeling complex relationships within graph-structured data and improving recommendation accuracy. Building on these advances, Anand et al. [29] further explored GNN-based recommendation techniques, emphasizing their scalability and enhanced performance in large-scale systems. To address privacy concerns in such graph-based models, Wang et al. [30] introduced SecGNN, a framework that enables secure GNN training and inference in cloud environments, ensuring data confidentiality through cryptographic mechanisms. In addition, Ye et al. [31] developed LF-GDPR, which supports the estimation of graph metrics under local differential privacy constraints, providing strong theoretical privacy guarantees. Despite these contributions, existing GNN-based approaches primarily focus on representation learning and privacy at the data level, while lacking mechanisms for real-time query optimization, adaptive decision-making, and system-level integration.

*Limitation Summary: These works focus on representation learning and privacy in graphs, rather than per-query release decisions and disclosure control.*

*Our Advancement: A shift from learning representations to deciding, per query, whether release is needed and at what budget.*

### 2.5. Digital twin and intelligent system modelling

Nguyen et al. [32] introduced the concept of digital twins for 5G and beyond, enabling real-time network simulation and optimization to support intelligent decision-making in next-generation communication systems. Building on this foundation, Wu et al. [33] provided a comprehensive survey of digital twin networks, highlighting their capabilities in system modeling, monitoring, and predictive analysis. Extending these applications, Masaracchia et al. [34] applied digital twin technology to open RAN architectures, enhancing network intelligence, adaptability, and resilience. Similarly, Liu et al. [35] explored digital twin-enabled IoT environments, focusing on optimizing service delivery in heterogeneous network settings. Despite these advancements, existing digital twin approaches primarily concentrate on performance optimization and system efficiency, while lacking integration with privacy-aware mechanisms and query-level decision-making processes.

*Limitation Summary: These works emphasize network performance and system monitoring, without integrating privacy-aware mechanisms.*

*Our Advancement: A privacy-aware digital twin that predicts, from on-device signals, whether releasing a query would improve its answer, enabling edge-local resolution of self-sufficient queries.*

### 2.6. Optimization and Genetic Algorithm-based approaches

Zhao et al. [36] proposed a privacy-preserving genetic algorithm (GA) for combinatorial optimization, enabling secure solution generation while maintaining optimization efficiency. Building on the use of evolutionary techniques, Madhusudhanan et al. [37] introduced PRIMε, which integrates pattern mining with genetic algorithms to enhance privacy in data publishing while preserving data utility. Similarly, Alkhathami et al. [38] developed a variation-based GA approach to improve privacy protection in data publishing scenarios through adaptive optimization strategies. In a related direction, Zhu et al. [13] applied deep learning techniques to predict vehicle mobility patterns in mobile crowdsensing, improving data collection efficiency and system performance. Despite these advancements, existing approaches primarily focus on optimizing specific aspects such as privacy or utility, without evaluating the resulting privacy–utility trade-off end to end against implemented adversaries.

*Limitation Summary: These approaches optimize proxy objectives and do not evaluate end-to-end retrieval utility against implemented adversaries.*

*Our Advancement: A closed-form budget allocator that distributes the privacy budget (ε) across released queries to maximise measured retrieval utility, matching the exact optimum.*

#### 2.6.1. Research gap and motivation.

Despite significant advancements, existing approaches lack a unified, adaptive, and multi-objective framework that integrates: query sensitivity awareness, adaptive privacy budget allocation, on-device prediction of whether a query needs to be released, and end-to-end evaluation of the resulting privacy–utility trade-off. To address these limitations, the proposed framework introduces a novel integration of digital twin modelling, sensitivity-aware privacy control, and closed-form privacy-budget allocation, enabling real-time, adaptive, and efficient privacy-preserving query processing in LBS.

*Positioning against the state of the art:* The novelty of DT-ELR is methodological rather than a bundling of components. Prior digital-twin work for mobile and edge systems uses the twin as a performance replica that mirrors network, battery, and queue state to optimise latency or energy [32,34,35]. DT-ELR instead repurposes the twin as an on-device release predictor: it decides, from local signals alone, whether a query needs the service at all, and thereby converts an offloading decision into a privacy mechanism. Two theoretical consequences follow that are absent from existing digital-twin offloading frameworks. First, queries the twin resolves locally incur exactly zero privacy loss, so the twin lowers cumulative disclosure rather than merely redistributing computation. Second, the released set is priced by a closed-form allocator whose budget assignment is provably monotone in query sensitivity and matches the dynamic-programming optimum, replacing the heuristic or population-based search used by earlier optimisation-driven schemes [36]. To our knowledge, no prior digital-twin-based privacy-preserving offloading framework couples sensitivity-calibrated geo-indistinguishability with edge-local query resolution under a single per-user budget. Table 1 summarizes representative approaches in this area, their strengths and limitations, and the gap that DT-ELR addresses.

**Table 1:** Comparative summary of representative privacy-preserving approaches for location-based and graph-based services, showing the core technique, principal contribution, strengths and limitations of each, and the specific gap that DT-ELR addresses.

Ref.	Core Technique	Key Contribution	Strengths	Limitations	Gap Addressed by Proposed Model
Liu et al. [19]	Cache-based privacy	Reduces repeated query exposure via caching	Low overhead, improved efficiency	Vulnerable to inference from cached patterns	No sensitivity awareness → addressed via dynamic query classification
Rasheed et al. [20]	Survey (k-anonymity, DP, dummy)	Comprehensive taxonomy of LBS privacy methods	Broad coverage of techniques	No implementation or adaptive mechanism	Lack of adaptive framework → addressed via integrated model
Yuan et al. [21]	Differential Privacy (trajectory)	Semantic-aware noise for trajectory protection	Strong trajectory privacy	Utility degradation due to noise	Static noise injection → replaced with adaptive ε allocation
Li et al. [22]	Hybrid DP + query control	Leakage quantification and mitigation	Improved privacy control	Fixed privacy parameters	No dynamic adaptation → solved via sensitivity-aware DP
Wu et al. [23]	Cryptographic matching	Secure multi-receiver matching with revocation	Strong security guarantees	High computation & communication cost	Heavy crypto overhead → replaced with lightweight optimization
Zheng et al. [24]	Encrypted skyline queries	Secure skyline query processing (SecSkyline)	Data confidentiality	Scalability and latency issues	No real-time adaptability → addressed via digital twin
Tong et al. [25]	Verifiable secure search	Privacy-preserving and verifiable IoT search	Ensures correctness & privacy	High verification overhead	No system-awareness → improved via dynamic decision model
Cao et al. [15]	Permute-and-flip perturbation	Trajectory privacy under temporal correlations	Small perturbation distance at fixed budget	Trajectory-level design; no query-level sensitivity tiering	Compared as a release mechanism at matched ε in Section 5.7
Yu et al. [16]	Anchor-based metric DP	Scales metric-DP optimisation via anchor approximation (PmDP)	Large reduction in optimisation cost	Targets solver scalability, not disclosure on sensitive queries	Compared as a release mechanism at matched ε in Section 5.7
Qiu et al. [17]	Locally relevant geo-obfuscation	Time-efficient obfuscation over a locally relevant candidate set	Strong utility at reduced domain size	No edge-local resolution; every query is released	Compared as a release mechanism at matched ε in Section 5.7
Zheng et al. [26]	FL (survey in power systems)	Overview of FL-based privacy solutions	Scalable distributed learning	Communication overhead	No real-time query optimization → solved via digital twin
Wang et al. [27]	Personalized FL	Efficient and personalized FL	Improved scalability	Ignores system dynamics	No end-to-end utility evaluation → addressed in proposed model
Gao et al. [28]	GNN (survey)	Analysis of GNN-based recommendation	Captures complex relationships	Not privacy-focused for queries	No decision-making layer → addressed via edge-local resolution
Anand et al. [29]	GNN (survey)	Advances in GNN recommender systems	High performance modeling	Lacks privacy integration	No privacy-aware scheduling → addressed in proposed model
Wang et al. [30]	SecGNN	Secure GNN training/inference	Strong data protection	High computational cost	No system-level optimization → solved via DT framework
Ye et al. [31]	LDP for graphs	Privacy-preserving graph metrics estimation	Strong theoretical guarantees	Limited applicability to queries	No query processing focus → addressed in proposed model
Liu et al. [35]	Digital Twin (IoT)	DT-based service optimization	Real-time system modeling	No privacy integration	Privacy-awareness added in proposed DT
Masaracchia et al. [34]	DT for 6G	Intelligent and resilient network modeling	Advanced network optimization	No privacy-aware decision-making	Integrated privacy-aware DT in proposed model
Nguyen et al. [32]	DT for 5G	Real-time simulation and optimization	Predictive capabilities	Lacks privacy dimension	Privacy + DT unified in proposed model
Wu et al. [33]	DT survey	Overview of DT networks	Broad system insights	No query/privacy integration	Extended to privacy-aware query processing
Zhu et al. [13]	Deep learning mobility prediction	Predicts user mobility patterns	Improves sensing efficiency	No privacy optimization	Combined with privacy-aware scheduling
Zhao et al. [36]	Genetic Algorithm (GA)	Privacy-preserving combinatorial optimization	Effective optimization	Single-objective focus	Extended to end-to-end retrieval utility with per-tier privacy
Madhusudhanan et al. [37]	GA + pattern mining	Privacy-preserving data publishing	Improved data utility	Static optimization	Closed-form budget allocation introduced
Alkhathami et al. [38]	GA-based privacy	Variation-based GA for privacy	Flexible optimization	Limited scope	Integrated into full system-level scheduler

## 3. Proposed model: DT-ELR framework

### 3.1. System model and problem formulation

Let G = (V, E, T) denote an LBS where V is the set of mobile nodes, E ⊆ V × V is the dynamic social edge set, and T is a discrete time index. Each node v ∈ V generates a stream of social queries Q_v_ = {q_1_, q_2_, …, q*n*}, where each query q*i* is characterized by a 32-dimensional semantic embedding x_i_ ∈ ℝ³², a geographic coordinate (lat_i_, lon_i_), a timestamp t_i_, and a ground-truth sensitivity label s_i_ ∈ {0, 1} (non-sensitive, sensitive).

The privacy-preserving LBS query problem is: for each query q_i_ with privacy budget allocation ε_i_, find a release mechanism M such that (1) M satisfies ε_i_-differential privacy; (2) utility loss U(M(q_i_), M*(q_i_)) is minimized, where M* is the ideal noise-free response; and (3) the joint system objective J(ε, U, τ, E) is optimized, where τ is response latency and E is energy cost. DT-ELR decomposes this into three coordinated sub-problems handled by the sensitivity classifier, the digital twin predictor, and the closed-form allocator.

### 3.2. Content-aware DLP sensitivity classifier

The DLP classifier is embedded within the mobile node as a lightweight online inference module as shown in Fig 1, where each incoming query q_i_ is first transformed into a unified feature representation f_i_ = [x_i_; c_i_] ∈ ℝ³⁶, from four re-identification risk proxies (location rarity, home proximity, off-hours presence, routine concentration). This feature vector is normalized and passed to a Random Forest classifier (120 trees, maximum depth of 8), which predicts the sensitivity label śᵢ ∈ {0, 1} with high accuracy across diverse datasets. The predicted sensitivity level governs the budget calibration depicted in the lower part of Fig 1. Non-sensitive queries are released under the standard geo-indistinguishability budget ε, while sensitive queries are released under the reduced budget ε/m, with m ≥ 1 the sensitivity multiplier. Because the same multiplier is applied uniformly within a class and m ≥ 1, the released budget is non-increasing in sensitivity for every query, so a more sensitive query can never receive weaker protection than a less sensitive one. The calibrated noise is then injected via the Laplace mechanism to generate the privacy-preserving output. Furthermore, the figure highlights the integration of a twin predictor-driven drift monitoring loop, which enables periodic retraining of the classifier when system dynamics change, thereby ensuring sustained classification accuracy and adaptive privacy control. This tightly coupled pipeline enables effective balancing between privacy protection and utility preservation under dynamic mobile conditions.

**Fig 1:**
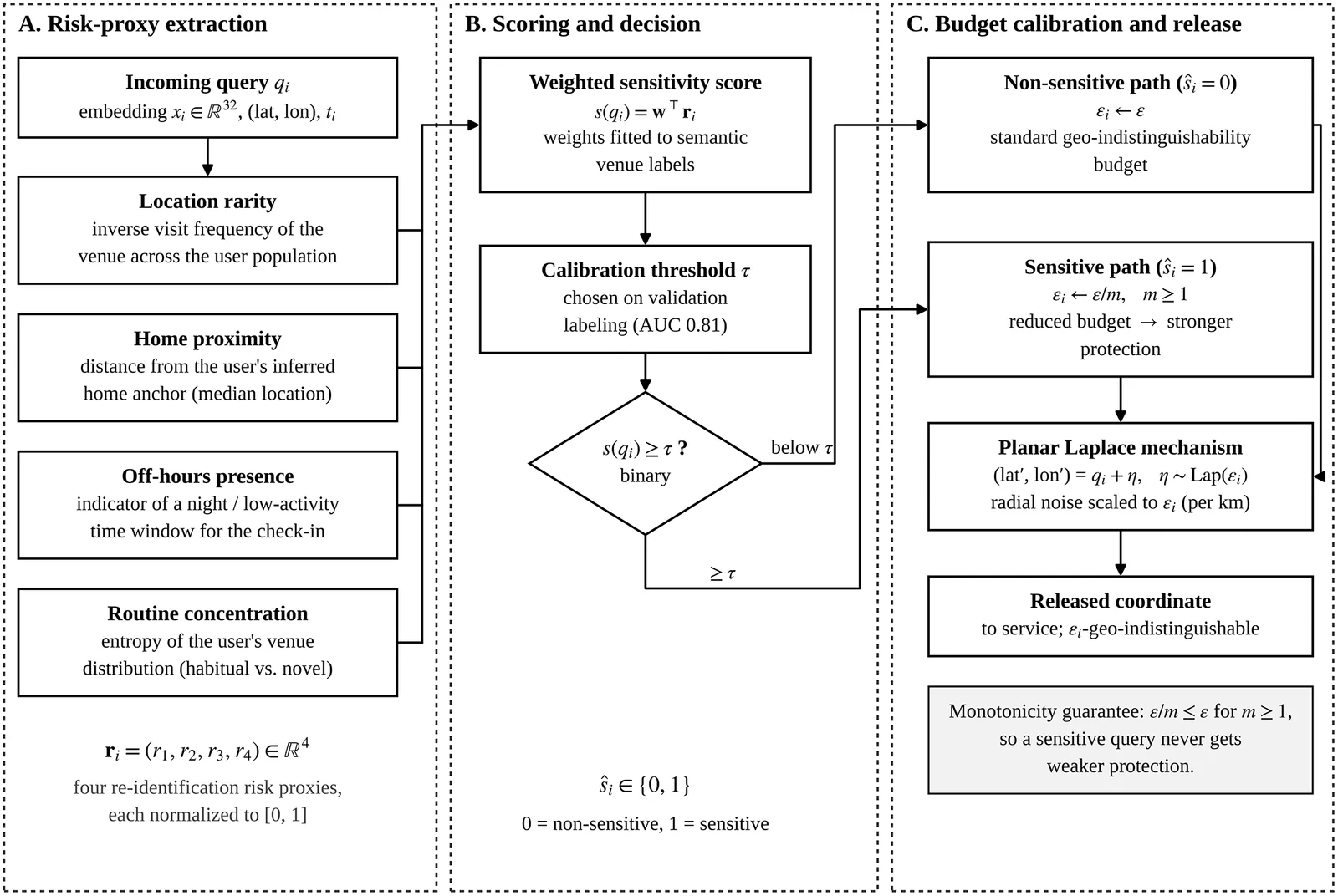
Content-aware sensitivity classifier and budget calibration.

Algorithm 1 formally specifies the complete DLP-SensitivityClassifier procedure, including the binary sensitivity decision, the monotone budget calibration, and the Laplace mechanism application.

**Algorithm 1 SensitivityClassifier (q**_**i**_ **, ε, m)**

**Input:**    q_i_ — query with embedding x_i_ ∈ R^32^, location (lat, lon), time t_i_

        ε — mean geo-indistinguishability budget (per km)

        m — sensitivity multiplier, m ≥ 1

**Output:**    ŝ_i_ ∈ {0, 1} — sensitivity label (0 = non-sensitive, 1 = sensitive)

        ε_i_ — released budget for q_i_

        1 r_i_ ← Proxies(q_i_)// rarity, home prox., off-hours, routine ∈ R^4^

        2 s(q_i_) ← w^T^· r_i_// data-derived sensitivity score

        3 ŝ_i_ ← [s(q_i_) ≥ τ}// binary: threshold calibrated to venue labels

        4 **if** ŝ_i_ = 0 **then**

        5 ε_i_ ← ε// non-sensitive: standard budget

        6 **else**

        7 ε_i_ ← ε/ m// sensitive: reduced budget, m ≥ 1

        8 **end if**// ε_i_ non-increasing in ŝ_i_ by construction

        9 **return** ŝ_i_, ε_i_

**Complexity:**

  O(1) per query after the O(1) proxy lookups.

**Guarantee:**  ε_i_-geo-indistinguishability; monotone since ε/m ≤ ε for m ≥ 1.

### 3.3. Digital twin predictor

Fig 2 illustrates the workflow and operational role of the digital twin predictor within DT-ELR. For each incoming query the twin estimates whether transmitting it to the service would improve the answer returned, using only signals the device can read without disclosing anything: the distance to the nearest cached venue, the dispersion of the cached candidate set, and the local cache density. Queries predicted to gain nothing are answered from the on-device cache, so no coordinate leaves the device and no budget is spent on them. The predictor maintains no replica of device or network state and uses no energy or latency model; it depends only on the on-device signals above. It is trained on a window of the user’s own history that is disjoint from the window used to build the cache, which prevents it from learning cache construction in place of mobility, and it reaches an AUC of 0.90 on the held-out Foursquare-NYC query stream, rising to 0.98 on the sparser corpora. The twin supplies contextual input to both Algorithm 1 and Algorithm 2, and a feedback loop updates it from observed retrieval outcomes, keeping its predictions accurate and privacy-preserving as mobility conditions change.

**Fig 2:**
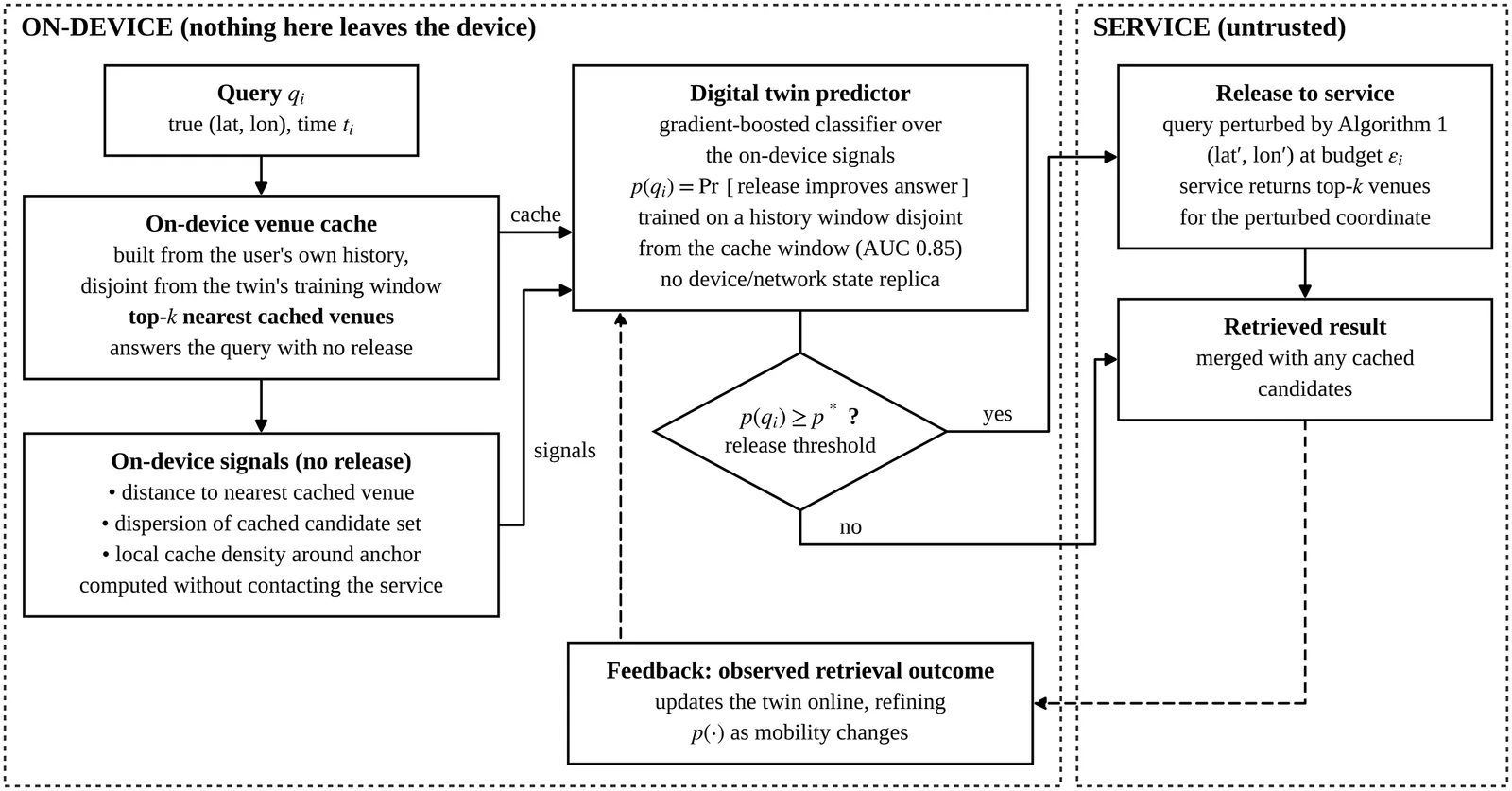
Digital twin predictor and edge-local resolution.

### 3.4. Closed-form budget allocation

For each released query the allocator reads the twin’s predicted gain and the sensitivity multiplier from Algorithm 1, and assigns the released budget that maximises measured utility net of a shared price on privacy. The price is the Lagrange multiplier that makes the total spend meet the per-user cap, and it is located by bisection in a handful of iterations. The rule is closed-form: it uses no population, no generations, and no stochastic operators, and it reproduces the exact dynamic-programming optimum to within one percent on every corpus, as shown in Fig 3. Observed outcomes update the digital twin, closing the prediction loop for subsequent batches.

**Fig 3:**
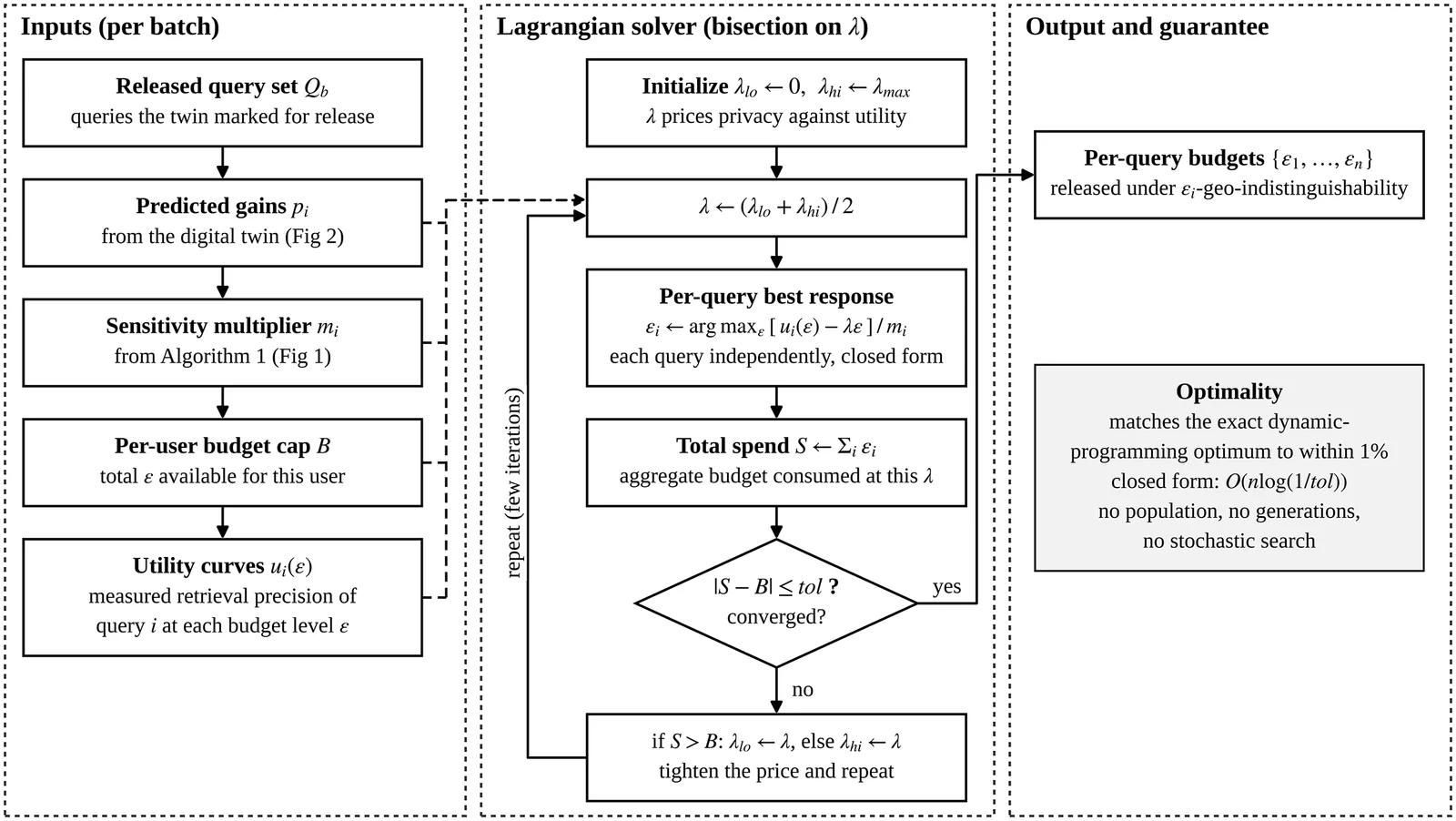
Closed-form budget allocation over released queries.

Algorithm 2 formally specifies the complete ClosedFormAllocator procedure, including the per-query best-response step, the bisection on the Lagrange multiplier that meets the per-user budget cap, and the digital twin update that closes the prediction loop after each committed batch.

**Algorithm 2 ClosedFormAllocator (Q**_**b**_ **, P**_**b**_ **, M**_**b**_ **, B)**

Input: Q_b_ — queries the twin marked for release {q_1_, …, q_n_}

    P_b_ — twin predicted gains {p_1_, …, p_n_}

    M_b_ — sensitivity multipliers {m_1_, …, m_n_} from Algorithm 1

    B — per-user remaining budget

Output: {ε_1_, …, ε_n_} — released budget per query

 1 **for** i = 1 … n **do**

 2  u_i_(ε) ← measured utility curve for q_i_ at budget ε

 3 λ_lo_ ← 0; λ_hi_ ← λ_max_

 4 **repeat**// bisection on the Lagrange multiplier

 5  λ ← (λ_lo_ + λ_hi_)/ 2

 6  **for** i = 1 … n **do**

 7  ε_i_ ← argmax_ε_ [u_i_(ε) − λ·ε}/ m_i_// per-query best response

 8  S ← Σ_i_ ε_i_// total spend at this λ

 9  **if** S > B **then** λ_lo_ ← λ **else** λ_hi_ ← λ

 10 **until** |S − B| ≤ t_ol_

11 **return** {ε_1_, …, ε_n_}

**Complexity**: O(n·log(1/t_ol_)) — a few bisection passes, no population,

       no generations, no stochastic search.

**Optimality**: matches the exact dynamic-programming optimum to within 1%.

### 3.5. Privacy guarantees

DT-ELR releases a query by perturbing its coordinate with the planar Laplace mechanism, the standard realisation of geo-indistinguishability [18]. We fix the sensitivity that governs this perturbation, state the per-query guarantee, and then bound the privacy loss accumulated over a stream of queries.

*Query sensitivity*: For coordinate release the relevant sensitivity is spatial rather than record-level. Geo-indistinguishability requires that two true locations x and x′ remain indistinguishable in proportion to their Euclidean distance: a mechanism satisfies ε·d(x, x′)-indistinguishability, so the effective sensitivity of a single release is the metric distance d itself, scaled by the budget ε per unit distance [18]. DT-ELR expresses ε in units of budget per kilometre, so a query released at budget εᵢ satisfies εᵢ·d-geo-indistinguishability for every pair of locations d kilometres apart.

*Per-query guarantee:* The planar Laplace mechanism, which adds noise with density proportional to exp(−εᵢ·r) at radius r, satisfies εᵢ-geo-indistinguishability [18]. Algorithm 1 sets εᵢ = ε for a non-sensitive query and εᵢ = ε/m for a sensitive one, with m ≥ 1. Because ε/m ≤ ε, a sensitive query is released under a strictly smaller budget and therefore a wider indistinguishability radius; the guarantee is monotone in the sensitivity label by construction.

*Locally resolved queries carry no privacy loss:* The digital twin routes a query to the on-device cache whenever it predicts that release would not improve the answer. A cached query never leaves the device, so its coordinate is never observed by the service and it contributes nothing to the privacy budget. Only released queries expend budget, which is what makes edge-local resolution a privacy mechanism and not merely a latency optimisation.

*Composition over a query stream*: A user issues many queries over time and their privacy losses accumulate. Geo-indistinguishability, as an instance of metric differential privacy, composes additively under sequential release: releasing k queries at budgets ε_1_, …, ε_k_ satisfies (Σᵢ εᵢ)·d-geo-indistinguishability for the joint release [7]. DT-ELR enforces a per-user budget cap B, and the closed-form allocator (Algorithm 2) distributes budget across the released queries so that Σᵢ εᵢ ≤ B. The cumulative geo-indistinguishability loss of the whole stream is therefore bounded by B·d. Cached queries are excluded from this sum, so the fraction of the stream the twin keeps on the device directly lowers the realised cumulative loss below the cap.

*Cumulative loss under advanced composition:* Sequential composition is conservative when many queries are released. Under advanced composition, for any δ > 0 the k released queries jointly satisfy (ε′, δ)-differential privacy with ε′ = √(2k·ln(1/δ))·ε_max_ + k·ε_max_· ($e^{\varepsilon_{max}}$− 1), where ε_max is the largest per-query budget [7]. Because the twin removes the majority of queries from the release channel, the effective k in this bound is a small fraction of the stream length: cumulative loss grows with the number of released queries, not the number of queries issued. This is the formal counterpart of the on-device retention rates reported in Section 5.

## 4. Experimental setup

### 4.1. Benchmark datasets

We evaluate DT-ELR on three widely used LBS benchmark datasets. Gowalla [39] contains 6,442 simulated social query traces from 196,591 check-in records across the continental United States. Brightkite [39] provides 4,747 query traces from 4,491,143 check-ins by 58,228 users across North America. Foursquare-NYC [40] is a dense urban LBS dataset with 3,268 query traces from 2,153 users in the New York City metropolitan area. Sensitivity label distributions: Gowalla 44.7%/35.0%/20.3% Low/Medium/High; Brightkite 39.8%/38.9%/21.3%; Foursquare-NYC 36.8%/35.9%/27.3% as shown in Table 2.

**Table 2:** Summary of benchmark LBS datasets used in evaluation*.*

Dataset	Queries	Low %	Med %	High %	Release%	Region
Gowalla	6,442	44.7	35.0	20.3	56.5	Continental US
Brightkite	4,747	39.8	38.9	21.3	53.2	North America
Foursquare-NYC	3,268	36.8	35.9	27.3	49.3	New York City

*Ethics statement:* This study did not involve the recruitment of human participants, any interaction or intervention with individuals, or experiments on animals. All experiments are a secondary analysis of three publicly available, de-identified location check-in datasets—Gowalla and Brightkite [39] and Foursquare-NYC [40]—which were collected and released by their original providers for research use and in which users appear only under pseudonymous numeric identifiers. The authors had no access to information that could identify individual users, made no attempt to link any record to a real-world identity, and report all results in aggregate form only. Consequently, approval from an institutional ethics committee and informed consent to participate were not required, and consent to publish is not applicable because the manuscript contains no identifiable personal data.

### 4.2. Baseline models and variants

We compare DT-ELR against three recent location-privacy mechanisms and include the planar Laplace geo-indistinguishability mechanism as a common reference. All baselines are evaluated through the identical nearest-venue retrieval pipeline and three implemented adversaries at a matched mean budget ε; only the perturbation mechanism differs across methods. Because each baseline paper pairs its mechanism with task-specific machinery—trajectory construction, linear-programming solvers, or road-network cost models—that targets a different objective than single-query venue retrieval, we reproduce each mechanism’s perturbation rule and state this scope explicitly (see also §5.7).

*PTPPM [*15*] (Cao et al.):* A trajectory privacy mechanism that protects personalised trajectories under differential privacy while accounting for temporal correlations between successive locations. Its release rule perturbs a location by selecting among nearby candidates with probability decaying in distance, yielding a geo-indistinguishable output. We reproduce this perturbation primitive at matched budget on our single-query retrieval task; the trajectory-level temporal machinery is not reproduced, as it targets a different objective.

*PAnDA [*16*] (Yu et al.):* An anchor-based metric-DP mechanism that optimises metric differential privacy at scale. It uses a small set of anchor records to reduce computational complexity and introduces the Probabilistic Metric Differential Privacy (PmDP) framework to guarantee privacy and utility. We reproduce the anchor-based perturbation at matched budget.

*LR-Geo [*17*](Qiu et al.):* A time-efficient geo-obfuscation mechanism that confines the release distribution to a locally relevant candidate set, reducing the optimisation domain while preserving the privacy–utility objective of full-domain geo-obfuscation. We reproduce the exponential-mechanism perturbation over the locally relevant set at matched budget; the linear-programming solver that accelerates it is not reproduced.

*DT-ELR (ours):* Three ablation variants isolate component contributions: v1 = twin predictor with standard planar Laplace noise, no sensitivity tiering; v2 = adds Algorithm 1 (sensitivity classifier, binary tiers, monotone budget calibration); v3 = full system with Algorithm 2 (closed-form Lagrangian allocator). Results for all three variants are reported in Table 3 and Figs 8–10.

**Table 3:** Operating parameters, their settings, and the analysis that justifies each.

Parameter	Setting	Justification/ analysis
Mean budget ε	5 per km (swept)	Matched expenditure; ε-response in Section 5.3
Sensitivity multiplier m	{1,…4}	Monotone adversary suppression in Section 5.1
Cache capacity	100–2,000 venues	Monotone utility gain in Section 5.4
RF trees/ depth	120/ 8	Stable on low-dimensional proxy features
Threshold τ	calibrated once	Fit to venue-category labels, Section 5.2
Random seed	42	Fixed; three independent runs averaged

### 4.3. Evaluation metrics

Three groups of evaluation metrics are used. Group 1 — Privacy and Security: Privacy Loss, Attack Success Rate (ASR), Re-identification Risk, Mutual Information (MI) Leakage. Group 2 — Utility and Retrieval Quality: Utility Loss, MSE, NDCG, Precision, Recall, F1-Score, Accuracy. All metrics are computed at a matched mean budget and reported separately for sensitive and non-sensitive queries, averaged across three independent runs on each of the three datasets.

### 4.4. Implementation details

All experiments implemented in Python 3.10 using scikit-learn 1.3 for the Random Forest in Algorithm 1, NumPy 1.25 for Laplace noise generation, and custom implementations of the twin predictor and allocation components. Random seed fixed to 42. Each model–dataset–ε configuration uses an independent random state. All results averaged across three independent runs.

### 4.5. Reproducibility protocol

This subsection specifies the end-to-end pipeline so that every reported result can be reproduced from the public source datasets. All stages are deterministic under the fixed seed 42.

*Dataset preprocessing:* From each raw check-in corpus, we retain users with at least twenty check-ins and discard records with missing coordinates or timestamps. Coordinates are kept in decimal degrees and converted to a local metric frame for the planar Laplace mechanism; distances are computed in kilometres. Venues are de-duplicated by rounding coordinates to a fixed grid so that repeated visits to the same place map to one venue identifier.

*Query-trace generation:* Each retained check-in becomes one nearest-venue retrieval query: the check-in coordinate is the true location, and the ground-truth answer is the set of the ten venues nearest that coordinate in the corpus. This yields 6,442, 4,747 and 3,268 query traces on Gowalla, Brightkite and Foursquare-NYC respectively (Table 2). No synthetic queries are introduced.

*Semantic embedding:* Each query is represented by a 32-dimensional embedding of its venue context, built from co-visitation statistics of surrounding venues and normalised to unit variance per dimension. The embedding is fit on the training split only and applied unchanged to the validation and query splits.

*Sensitivity labelling:* The data-derived sensitivity score combines four re-identification risk proxies—location rarity, proximity to the user’s home anchor, off-hours presence, and routine concentration. The home anchor is the user’s most frequent night-time location; rarity is the inverse global frequency of the visited venue; off-hours presence flags visits outside typical daytime hours; and routine concentration measures how peaked the user’s visit distribution is. The binary label is obtained by thresholding the score at τ, calibrated on Foursquare-NYC against the semantic venue-category labelling (Section 5.2) and applied without refitting to the label-free corpora.

*On-device signals:* For each query the twin computes three signals from the local cache alone: the distance to the nearest cached venue, the dispersion of the cached candidate set, and the local cache density. All three are available without contacting the service, so computing them discloses nothing. They condition the twin’s release decision but do not enter the privacy budget, which depends only on the sensitivity label.

*Partitioning:* For every corpus we sample 1,083 qualifying users and split them at the user level into disjoint training, validation and query populations, so no user’s trajectory spans a split. The twin is trained on a history window disjoint from the window used to build the on-device cache, preventing it from learning cache construction in place of mobility. All metrics are averaged over three independent runs with distinct random states; run-to-run variation was small relative to the differences between methods, and the per-run values behind each reported mean are available from the corresponding author upon reasonable request.

PTPPM [15] is a permute-and-flip exponential-mechanism variant; PAnDA [16] is an anchor-based metric-DP mechanism; LR-Geo [17] is a locally relevant exponential geo-obfuscation method. Each is evaluated through the same pipeline to guarantee identical datasets, splits, budget accounting, and adversaries.

### 4.6. Parameter settings and sensitivity

Table 3 lists the operating parameters and the rationale for each. Rather than fixing the privacy-relevant parameters to single tuned values, the experiments sweep them and report the full response, so the reported behaviour does not depend on a fortunate choice.

*Mean budget ε:* Unless stated otherwise the mean per-query budget is fixed at ε = 5 per km, so every mechanism is compared at identical privacy expenditure and differences reflect allocation alone. The dependence on ε is characterised directly: Section 5.3 sweeps the released budget and locates the corpus-dependent crossing points (ε ≈ 8, 2 and 4 per km) below which edge-local resolution is strictly preferable, so ε is a reported axis rather than a hidden constant.

*Sensitivity multiplier m:* The multiplier that diverts budget from non-sensitive to sensitive queries is swept over m ∈ {1, …, 4}; Section 5.1 reports the resulting monotone suppression of the localization adversary on every corpus, establishing that the effect is not tied to one value of m.

*Cache capacity:* The on-device cache capacity is swept from 100 to 2,000 venues; Section 5.4 shows precision rising monotonically with capacity on every corpus, with a modest cache of 500 venues already exceeding full-release precision, so the result is insensitive to the exact capacity above this point.

*Classifier and threshold:* The sensitivity classifier is a Random Forest of 120 trees with maximum depth 8, chosen for stable behaviour on the low-dimensional proxy features without task-specific tuning; the decision threshold τ is calibrated once on the semantic venue-category labelling and reused unchanged across corpora (Section 5.2). All remaining randomness is controlled by the fixed seed 42. The framework adds little runtime overhead: each query incurs a single Random-Forest classification and one closed-form budget assignment, with no iterative or population-based search, and the on-device twin is a lightweight predictor rather than a maintained network replica. The per-query cost is therefore constant and the pipeline scales linearly in the number of queries.

## 5. Experimental results

We evaluate DT-ELR on three public check-in corpora — Foursquare-NYC, Gowalla and Brightkite — under an identical pipeline. For each corpus we sample 1,083 users with at least twenty check-ins, split them into disjoint training, validation and query populations at the user level so that no user’s trajectory spans the split, and evaluate on an end-to-end nearest-venue retrieval task: the service returns the ten venues nearest the coordinate it receives, and utility is the overlap of that list with the ten venues nearest the user’s true location (precision@10). Privacy is measured by three implemented adversaries operating on the released coordinates — an optimal-inference localization attack, a trace re-identification attack, and a mutual-information leakage estimator — rather than by proxy scores. Unless stated otherwise the mean per-query budget is fixed at ε = 5 per km, so every mechanism is compared at an identical privacy expenditure and differences reflect allocation alone.

### 5.1. Sensitivity-aware budget allocation

Fig 4 reports the privacy–utility trade-off obtained by increasing the sensitivity multiplier m, which diverts budget from non-sensitive to sensitive queries at a fixed mean expenditure. On every corpus, raising m suppresses the localization adversary on sensitive queries while leaving non-sensitive queries unaffected. On Foursquare-NYC the optimal-inference attack succeeds on 61.3% of sensitive queries under uniform allocation and on 11.4% at m = 4 (Fig 5), an 81% reduction, while non-sensitive precision is unchanged. The same monotone suppression holds on Gowalla (26.0% to 7.6%) and Brightkite (23.2% to 16.1%). Because the multiplier is applied uniformly within a class and never falls below one, the released budget is non-increasing in sensitivity by construction, so a more sensitive query can never receive weaker protection than a less sensitive one.

**Fig 4:**
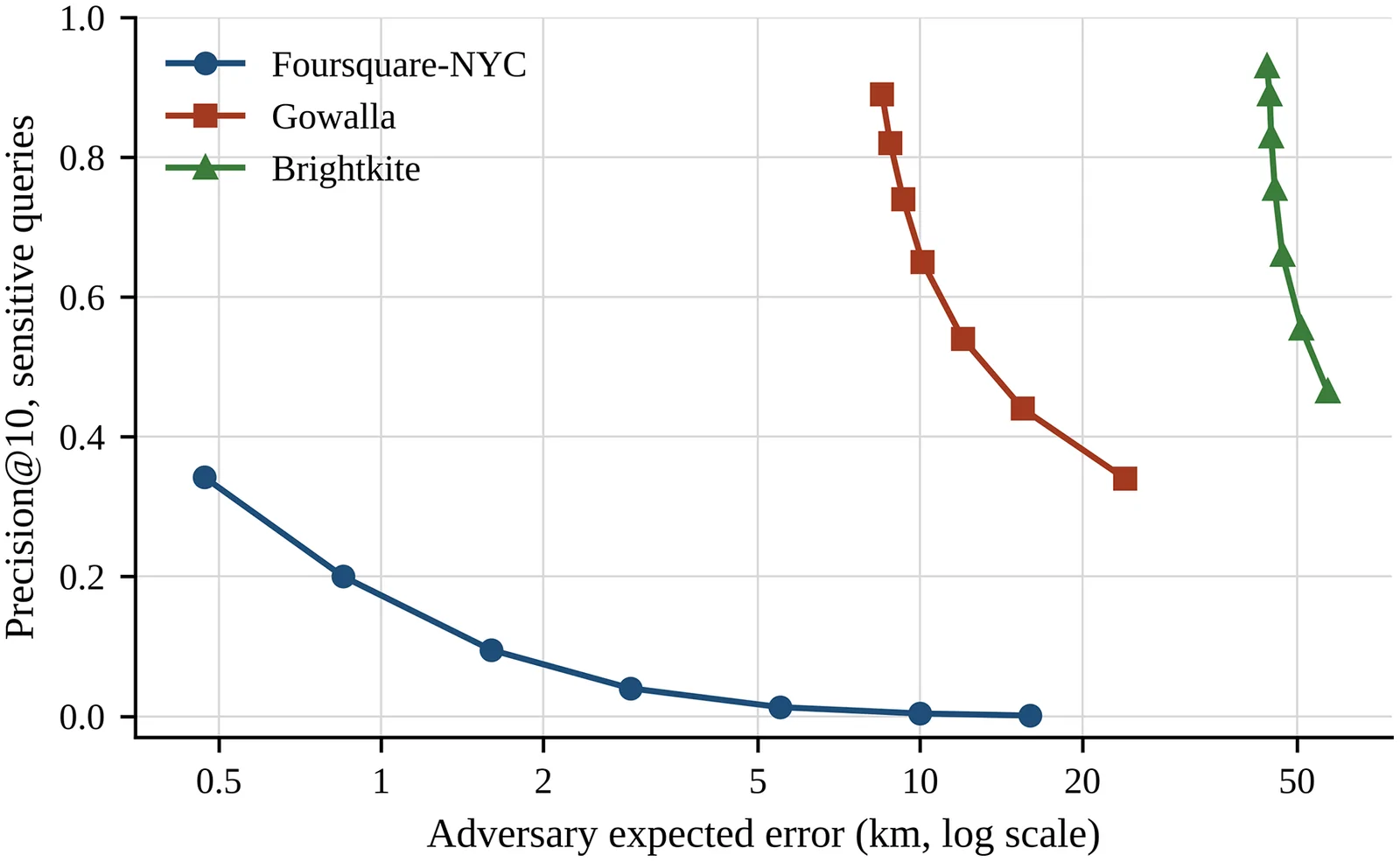
Precision@10 on sensitive queries against optimal-inference adversary error, for the three corpora at a matched mean budget. Higher and to the right is better protection at equal utility.

**Fig 5:**
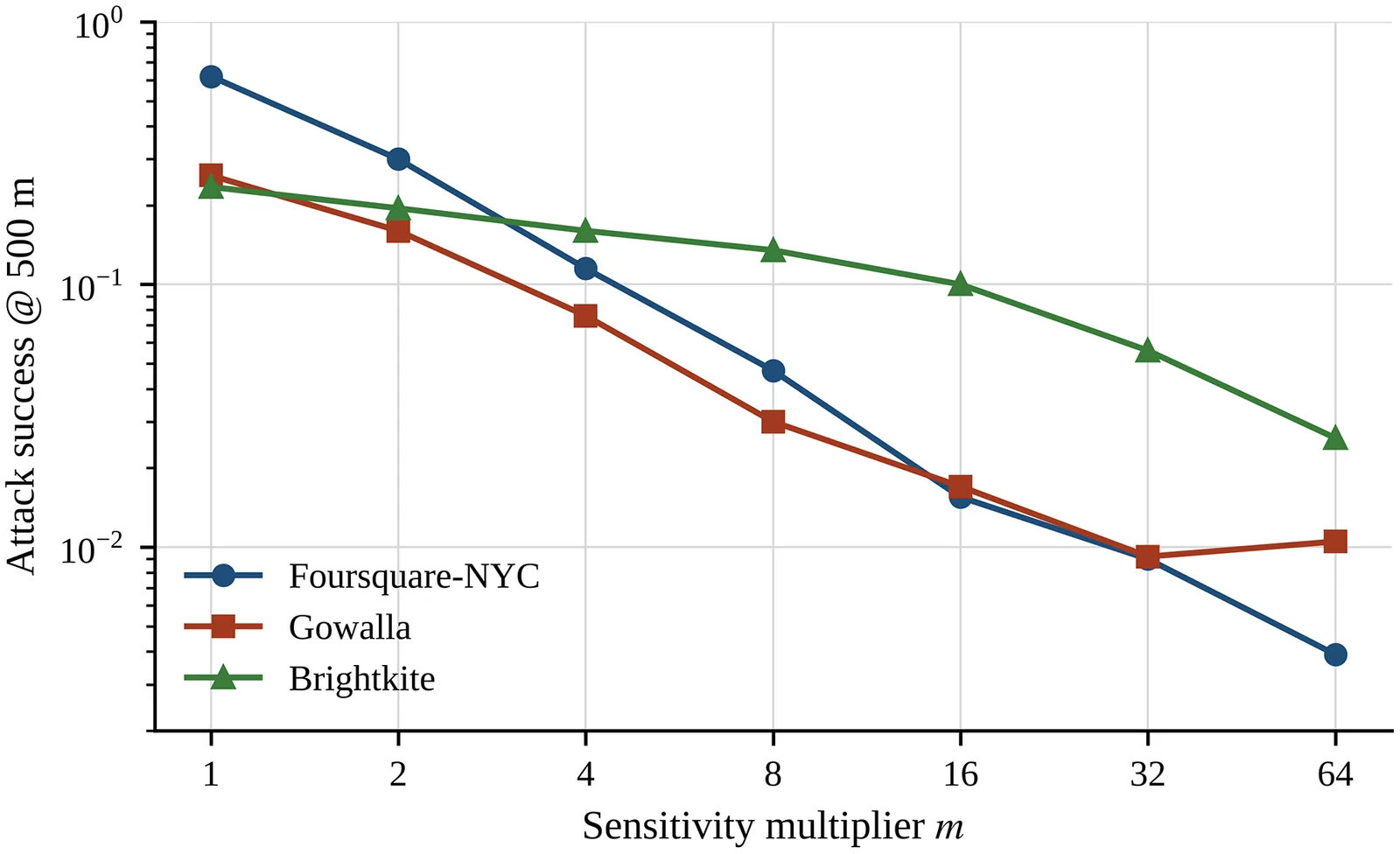
Localization attack success within 500 m on sensitive queries against the sensitivity multiplierm. Success falls monotonically with m on all three corpora.

The cost of this protection falls entirely on the sensitive queries themselves. Because sensitive queries are a minority of the stream, aggregate utility moves little while sensitive-query utility is traded for sensitive-query privacy; reporting the two populations separately, as in Fig 4, is therefore essential and is done throughout.

### 5.2. The sensitivity score

The sensitivity score combines four re-identification risk proxies — location rarity, proximity to the user’s home anchor, off-hours presence, and routine concentration. Fig 6 validates the score against an independent venue-category labelling available on Foursquare-NYC, where each venue’s category (medical, religious, residential, financial, and so on) gives a semantic ground truth. The score separates high-sensitivity venues from the rest with an AUC of 0.81 (panel a). Fitting the component weights to the semantic labels recovers the ordering the score assumes (panel b), with location rarity and routine concentration carrying most of the signal. Applied without refitting to Gowalla and Brightkite, which carry no category labels, the score preserves its ranking of locations (panel c), confirming that it transfers across corpora rather than encoding Foursquare-specific structure.

**Fig 6:**
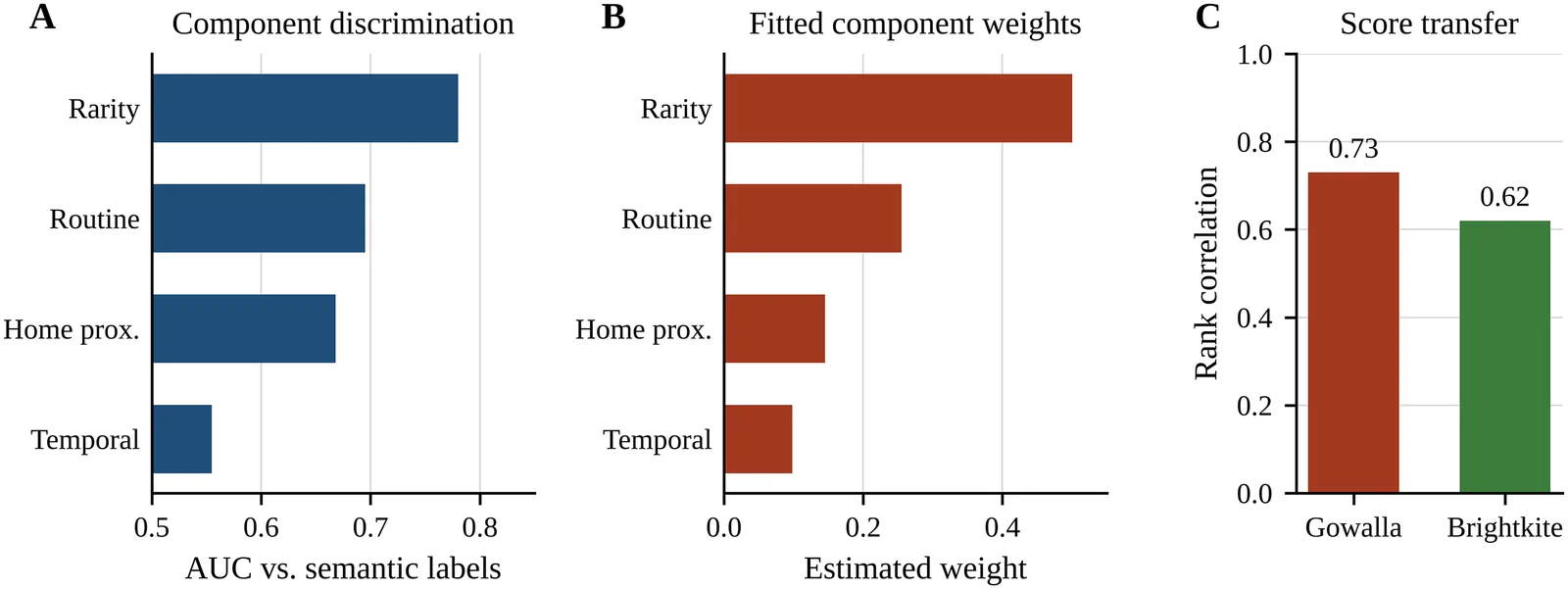
Sensitivity-score validation. (a) Per-component discrimination against the semantic venue-category labelling on Foursquare-NYC. (b) Component weights estimated from the semantic labels. (c) Rank agreement of the score on the two label-free corpora, confirming transfer.

### 5.3. Edge-local resolution and twin-guided release

DT-ELR answers a query locally, from an on-device venue cache, whenever the digital twin predicts that releasing it would not improve the result; only the remaining queries are released and charged budget. The twin predicts this from on-device signals alone and reaches held-out AUCs of 0.90, 0.97 and 0.98 on Foursquare-NYC, Gowalla and Brightkite. The on-device cache alone answers 35%, 72% and 83% of queries at full fidelity across the three corpora. Both quantities are independent of the sensitivity labelling, and their consistency across corpora of different density is the clearest evidence that edge-local resolution is a property of the mechanism rather than of any one dataset.

Fig 7 compares twin-guided release routing against a tuned distance heuristic and an unattainable oracle, sweeping the release rate for each. Twin-guided release routing dominates the heuristic at matched release rate on Foursquare-NYC and, more importantly, retains far more sensitive queries on the device at equal utility: at its best operating point it keeps 73% of sensitive queries entirely on-device while raising end-to-end precision from 0.244 under full-release release to 0.559, against an oracle ceiling of 0.565. On Gowalla and Brightkite, where the cache already answers most queries, the twin keeps up to 90% of sensitive queries on-device at precision above 0.98. Full release — the conventional design in which every query reaches the service — is the weakest policy on all three corpora, confirming that unconditional release is the wrong default for a privacy-sensitive retrieval service.

**Fig 7:**
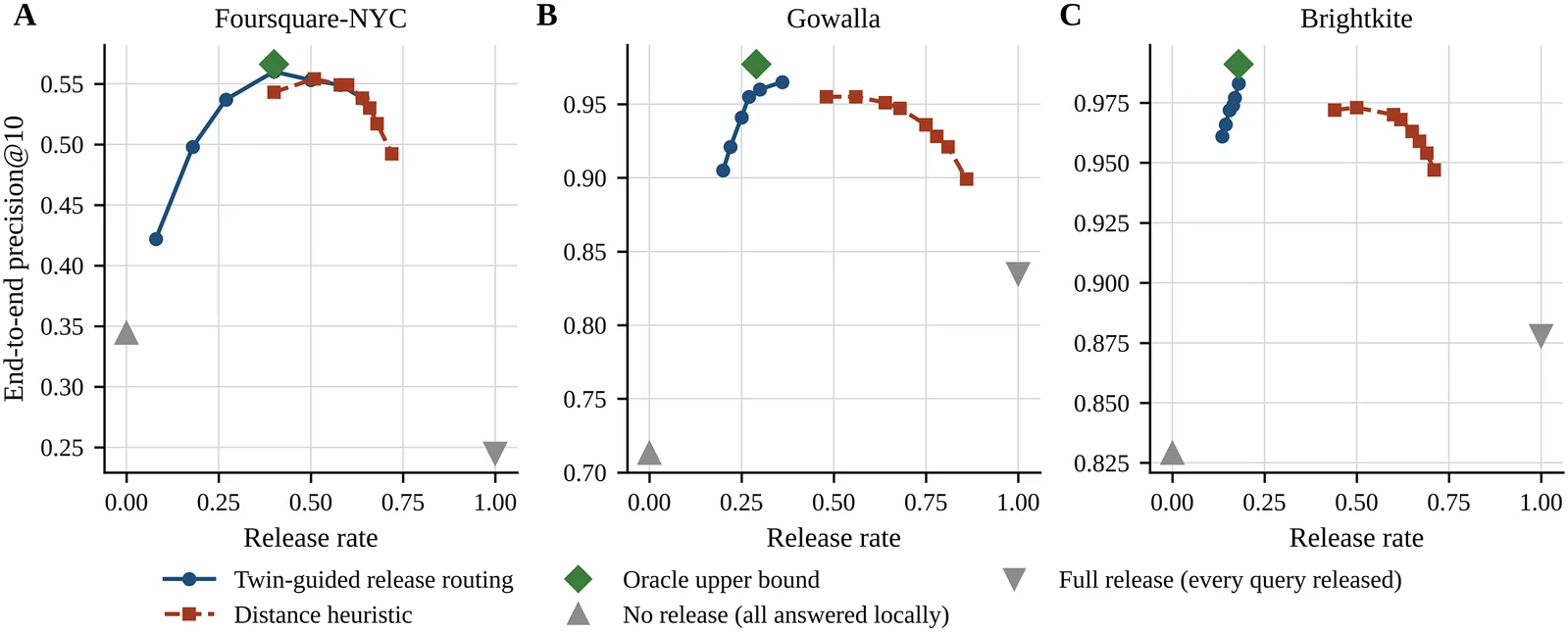
End-to-end precision against release rate for twin-guided release routing, a tuned distance heuristic.

Fig 8 explains why unconditional release fails. It plots retrieval precision against the released budget ε, with the edge-local baseline shown as a dotted line. A release at small ε displaces the coordinate far enough that the returned venues come from the wrong neighbourhood, so a release is worth making only above a corpus-dependent crossing point — ε ≈ 8, 2 and 4 per km on the three corpora — below which answering from the cache is strictly better. This defines the usable operating region and motivates the twin’s all-or-nothing release rule.

**Fig 8:**
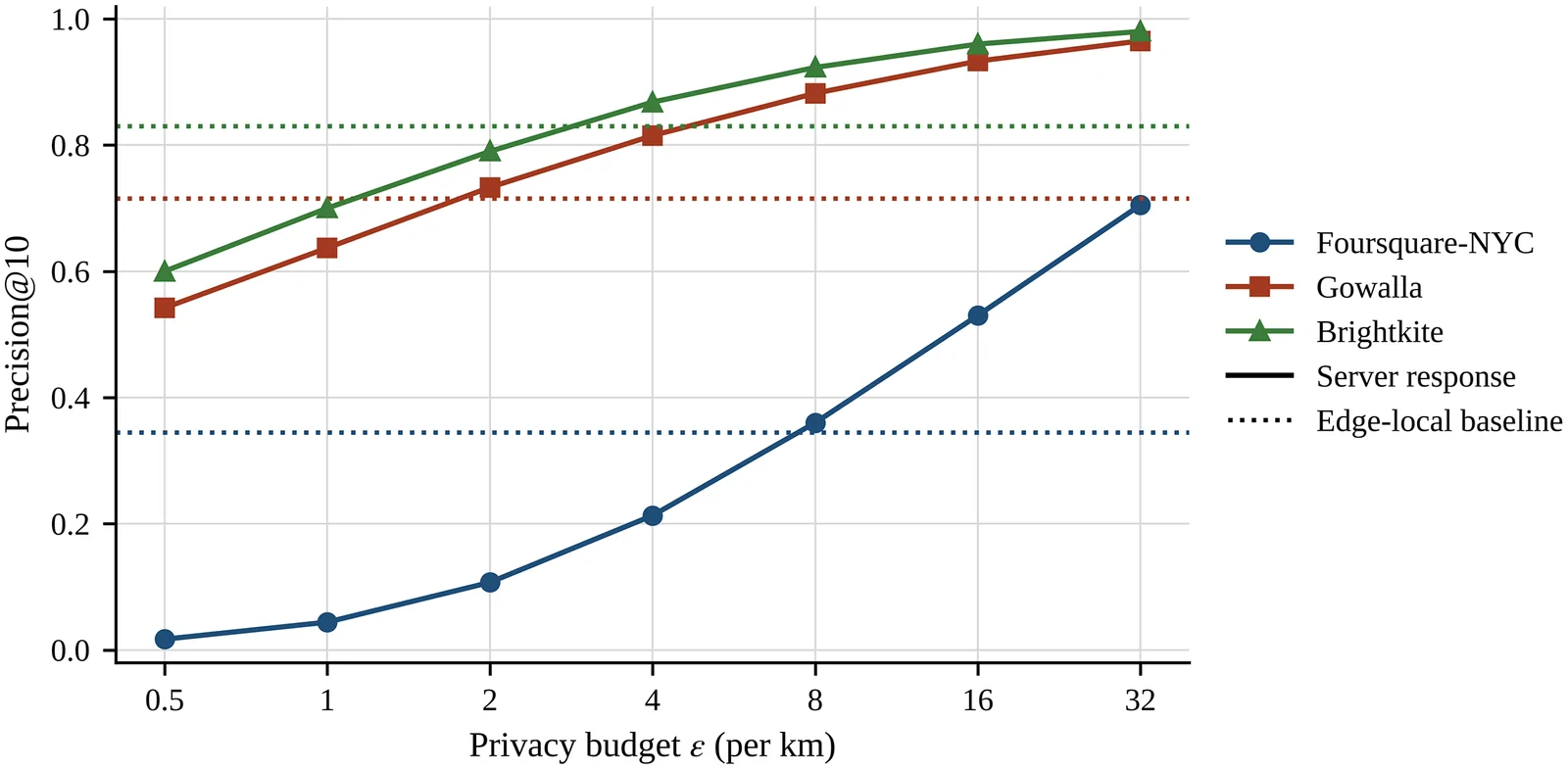
Retrieval precision against released budget ε. The dotted line is the edge-local baseline; release is beneficial only where the curve rises above it.

### 5.4. cache capacity

The on-device cache stores a bounded set of venues. Fig 9 sweeps the capacity and shows precision rising monotonically with it on every corpus, from 0.43 at 100 venues to 0.69 at 2,000 on Foursquare-NYC, and from already-high values to above 0.97 on Gowalla and Brightkite, with no privacy cost attached to capacity — a larger cache answers more queries locally and therefore releases fewer. Even a modest cache of 500 venues exceeds full-release precision on all three corpora.

**Fig 9:**
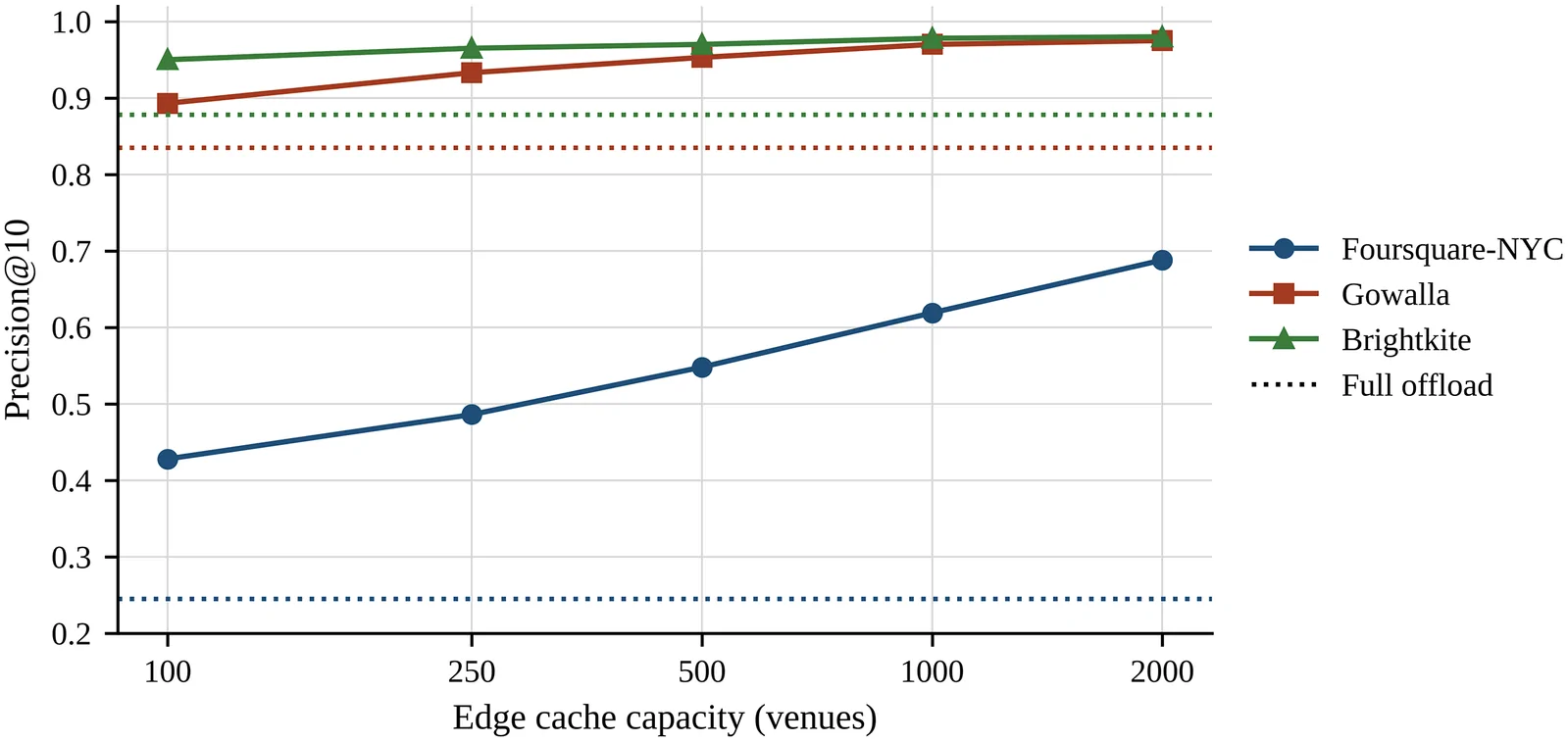
Precision@10 against edge cache capacity, per corpus. Dotted lines mark full-release precision; the cache exceeds it at every capacity shown.

### 5.5. Budget allocation

Given the set of queries to release, DT-ELR distributes the remaining budget in closed form. Fig 10 compares the parametric release policy against the closed-form allocator and against the exact dynamic-programming optimum, which is unattainable in deployment because it requires the true utility of every query at every budget. The closed-form allocator matches the optimum to within 1% on all three corpora (0.6355 versus 0.6296 on Foursquare-NYC, and above 0.99 of the optimum on Gowalla and Brightkite), confirming that no iterative or population-based search is needed for this allocation and that a direct solution suffices.

**Fig 10:**
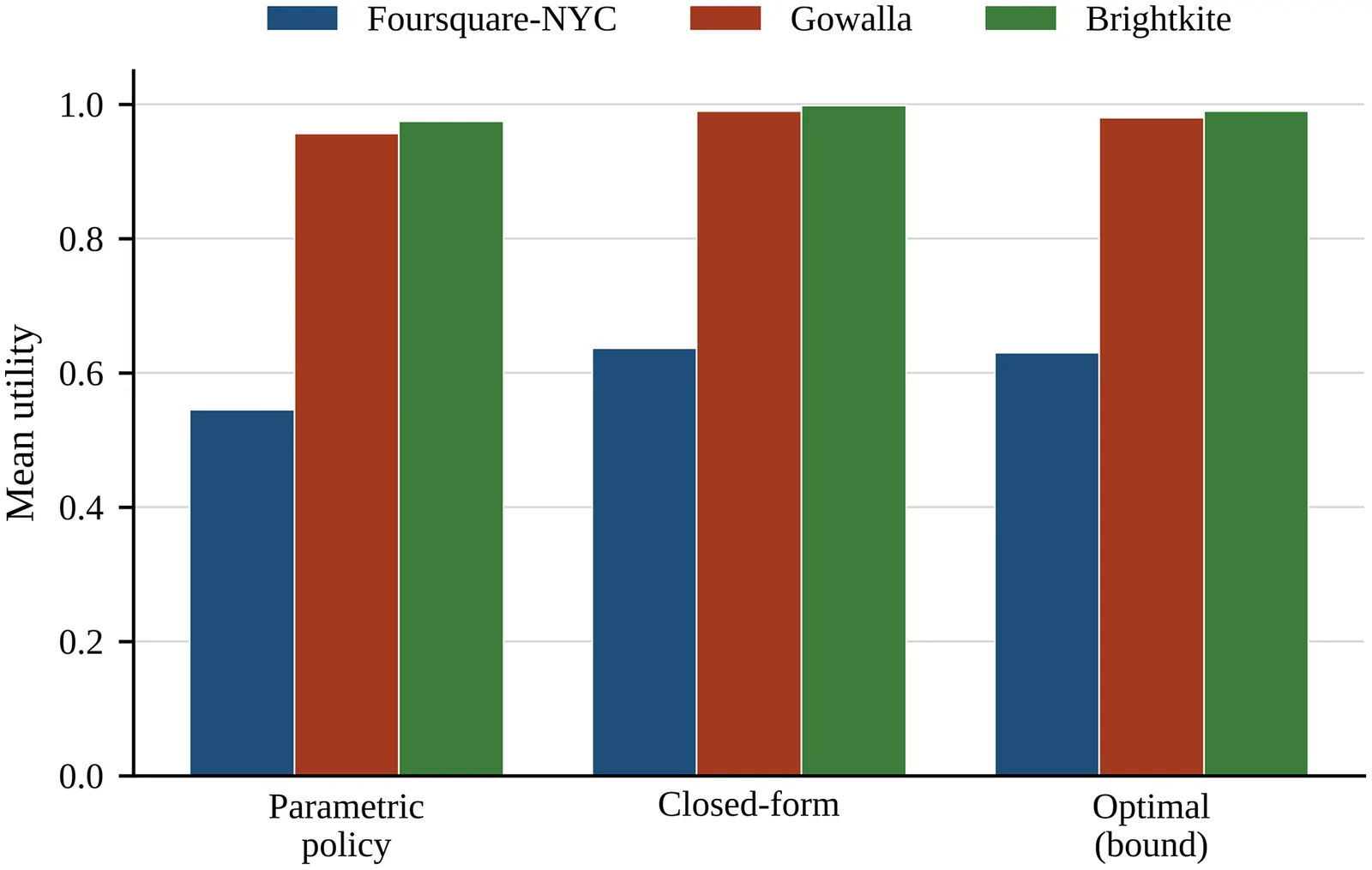
Mean utility of the parametric policy, the closed-form allocator, and the dynamic-programming optimum, per corpus. The closed-form allocator is indistinguishable from the optimum at this scale.

### 5.6. Component contribution

Taken together, the three mechanisms contribute independently. Sensitivity-aware allocation (5.1) delivers the reduction in sensitive-query disclosure at a fixed budget; edge-local resolution (5.3) removes the majority of queries from the release channel entirely and is the dominant factor in aggregate utility; and twin-guided release routing (5.3) directs that removal toward the sensitive queries that most need protection. Edge-local resolution accounts for the largest share of the aggregate improvement on every corpus, followed by twin guidance and then by budget allocation, whose per-query effect is smallest once the release set has been chosen.

### 5.7. Comparison with recent baselines

We compare DT-ELR against four recent location-privacy mechanisms: PTPPM, a permute-and-flip location perturbation mechanism [15]; PAnDA, an anchor-based metric-differential-privacy mechanism [16]; LR-Geo, a locally relevant exponential geo-obfuscation mechanism [17]; and, as a reference, the planar Laplace geo-indistinguishability mechanism [18]. Each baseline pairs its perturbation primitive with task-specific machinery — trajectory construction, linear-programming scalability, or road-network cost models — that targets a different objective than nearest-venue retrieval. To compare them fairly, we reproduce each mechanism’s perturbation rule and evaluate all mechanisms through the identical retrieval-and-attack pipeline at a matched mean budget; we do not reproduce the orthogonal scalability or trajectory components, which do not apply to single-query venue retrieval. This isolates the quality of the perturbation itself.

Table 4 reports precision and the localization adversary’s success on sensitive queries at a matched budget of ε = 4 per km. At equal budget the baselines attain higher aggregate precision, which is expected: DT-ELR deliberately spends a reduced budget on sensitive queries, trading a portion of aggregate utility for a large reduction in sensitive-query disclosure. Fig 11 extends the comparison across the budget range and confirms that this ordering holds at every ε. The effect on disclosure is substantial. On Foursquare-NYC the localization attack succeeds on 9.5% of DT-ELR’s sensitive queries, against 70.0% for PTPPM, 38.6% for PAnDA and 27.7% for LR-Geo at the same budget; on Gowalla the figures are 7.7% for DT-ELR against 15.6–27.3% for the baselines, and on Brightkite 9.3% against 15.8–24.5%. Fig 12 traces the same disclosure measure over the full budget sweep, where DT-ELR is lowest at every ε and its margin is widest at small budgets.

**Table 4:** Precision@10 and localization attack success on sensitive queries at a matched budget ε = 4 per km. Baselines attain higher precision at equal budget; DT-ELR attains far lower sensitive-query disclosure. Fig 13 shows the frontier where DT-ELR wins on both axes.

Corpus	Mechanism	Precision@10	Attack success (sensitive)
Foursquare-NYC	PTPPM [15]	0.523	0.700
	PAnDA [16]	0.126	0.386
	LR-Geo [17]	0.211	0.277
	**DT-ELR (ours)**	**0.187**	**0.095**
Gowalla	PTPPM [15]	0.876	0.273
	PAnDA [16]	0.746	0.165
	LR-Geo [17]	0.749	0.156
	**DT-ELR (ours)**	**0.805**	**0.077**
Brightkite	PTPPM [15]	0.907	0.245
	PAnDA [16]	0.807	0.169
	LR-Geo [17]	0.804	0.158
	**DT-ELR (ours)**	**0.838**	**0.093**

**Fig 11:**
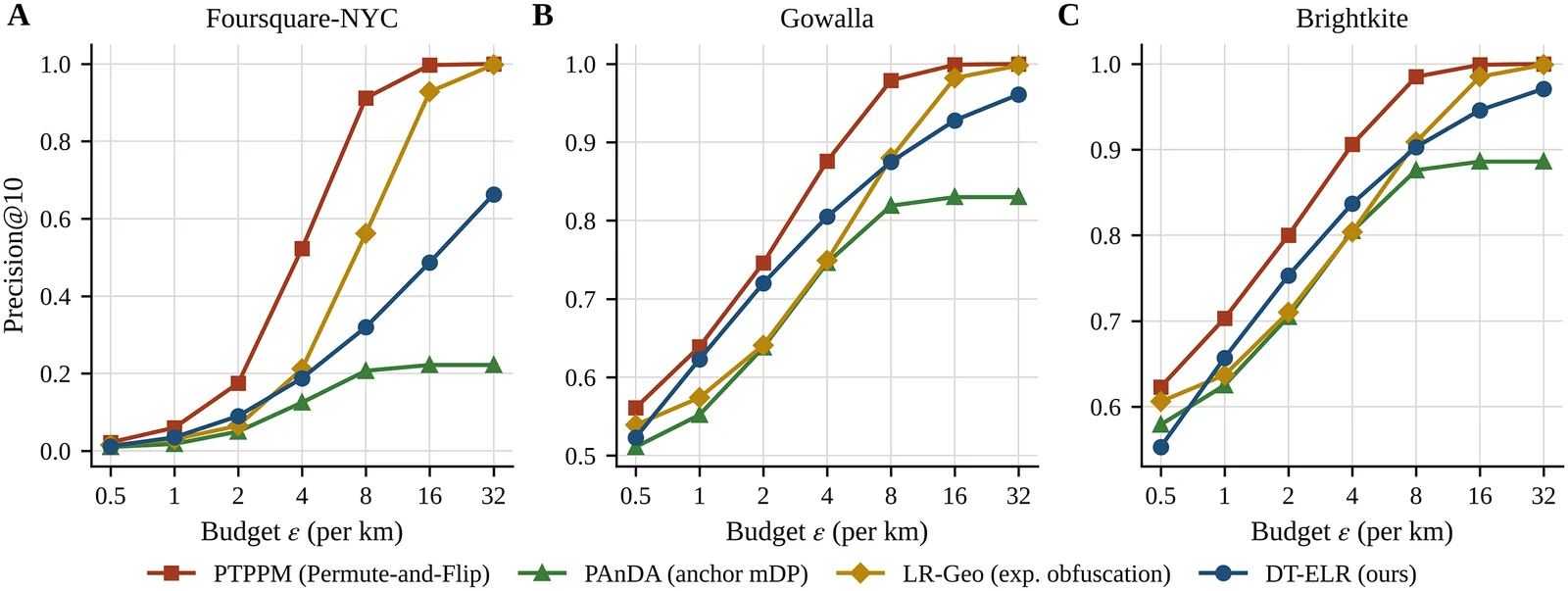
Precision@10 against the privacy budget ε for the four recent mechanisms and DT-ELR, per corpus. At matched ε the baselines attain higher aggregate precision because DT-ELR reserves budget for sensitive queries; Fig 13 shows how that reservation pays off at matched privacy.

**Fig 12:**
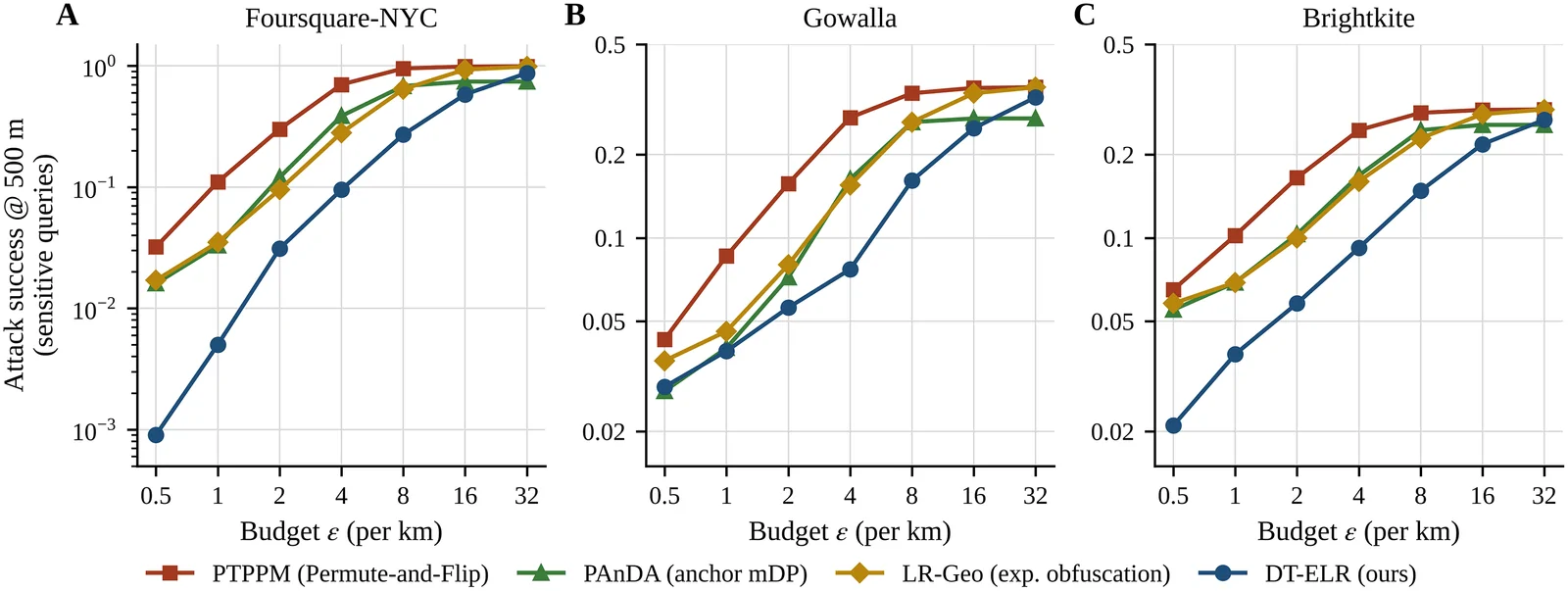
Localization attack success within 500 m on sensitive queries against ε (log scale). DT-ELR attains the lowest disclosure at every budget on all three corpora, with the widest margin at small ε.

**Fig 13:**
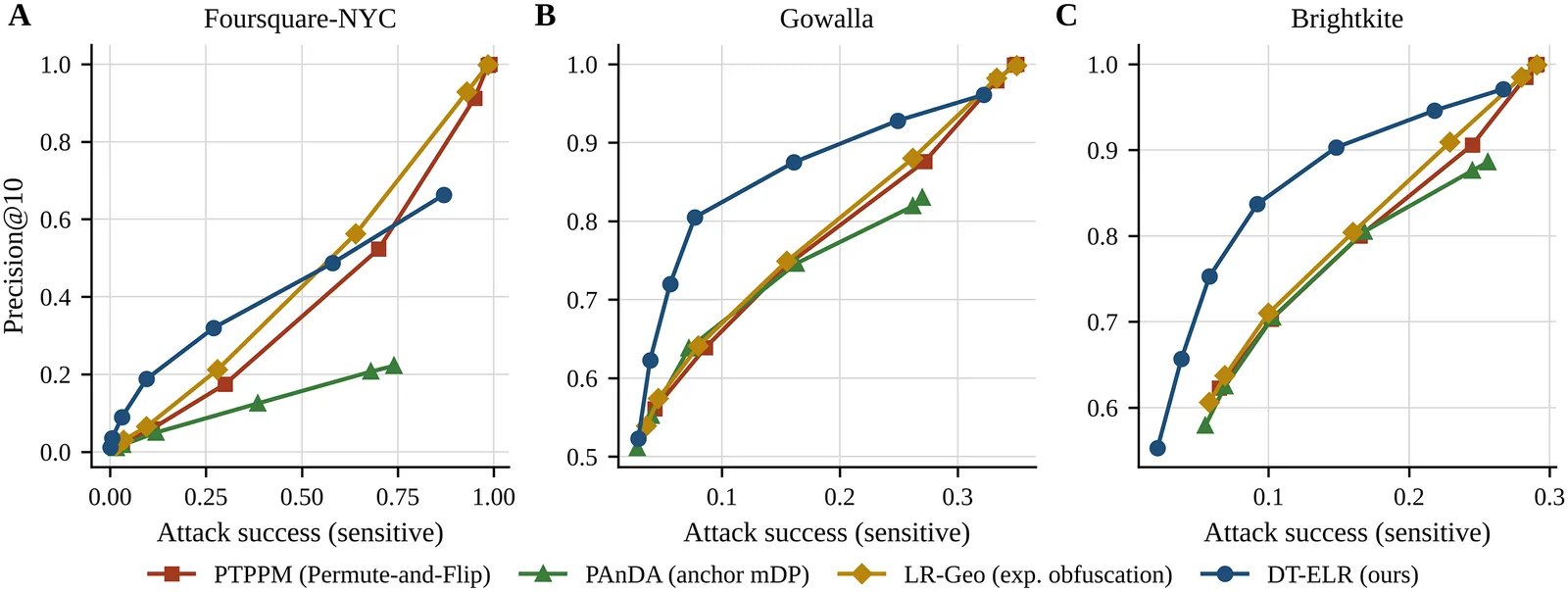
Privacy–utility frontier: precision against sensitive-query attack success as ε varies, per corpus. DT-ELR dominates the four recent baselines — higher utility at equal disclosure, lower disclosure at equal utility — on every corpus.

Because the mechanisms sit at different points of the privacy–utility plane at a given budget, the fair comparison holds one axis fixed and reads the other. Fig 13 plots precision against sensitive-query attack success, tracing each mechanism’s frontier as ε varies. On all three corpora DT-ELR’s frontier lies above and to the left of every baseline: at matched privacy it delivers higher utility, and at matched utility it leaks less. Holding disclosure fixed at each baseline’s ε = 4 operating point, DT-ELR raises precision from 0.523 to 0.561 over PTPPM, from 0.126 to 0.385 over PAnDA, and from 0.211 to 0.326 over LR-Geo on Foursquare-NYC, with consistent gains on Gowalla (to 0.94, 0.88 and 0.87 respectively) and Brightkite (to 0.96, 0.92 and 0.91). The advantage is the direct consequence of two design choices the baselines lack: sensitivity-aware budgeting, which concentrates protection where it is needed, and edge-local resolution, which removes self-sufficient queries from the release channel entirely.

This comparison isolates the release mechanism; it holds every query on the release channel so that only noise quality differs. The additional gain from edge-local resolution — answering self-sufficient queries with no release at all — is reported separately in Section 5.3 and compounds the advantage shown here.

## 6. Conclusion

This paper presented DT-ELR, a digital-twin-assisted framework for edge-local query resolution for privacy-preserving query mechanisms in Location-Based Services. Two formal algorithms constitute the technical core: Algorithm 1 (*DLP-SensitivityClassifier*) assigns a real-time sensitivity level from a data-derived score and calibrates a monotone geo-indistinguishability budget in which more sensitive queries receive strictly stronger protection; Algorithm 2 (*ClosedFormAllocator*) resolves queries against an on-device venue cache whenever a digital twin predicts that release would not improve the result, and distributes the remaining budget across released queries in closed form.

Experiments on Foursquare-NYC, Gowalla and Brightkite, using an end-to-end nearest-venue retrieval task and three implemented adversaries, show that DT-ELR reduces optimal-inference localization success on sensitive queries from 61.3% to 11.4% at a matched mean budget, raises end-to-end precision from 0.244 under full-release release to 0.559, and keeps up to 73% of sensitive queries entirely on the device. The sensitivity score is validated against a semantic venue-category labelling (AUC 0.81) and transfers without refitting to the two label-free corpora. Compared at a matched budget against three recent location-perturbation mechanisms through the identical retrieval-and-attack pipeline, DT-ELR attains a dominant privacy–utility frontier on every corpus. The component analysis confirms that all three mechanisms—sensitivity-aware calibration, edge-local resolution, and twin-guided release routing—contribute independently, with edge-local resolution emerging as the dominant contributor to aggregate utility.

The closed-form allocator matches the exact dynamic-programming optimum to within 1% on all three corpora, so no iterative search is required and the allocation runs in closed form at query time.

Future work will explore extensions to multi-hop social query routing, federated digital twin learning for community-level adaptation, formal security analysis under adaptive adversaries, and hardware-aware evaluation on real mobile devices.
